# bpRNA-CosMoS: a robust and efficient RNA structural comparison method using k-mer based cosine similarity

**DOI:** 10.1093/bioinformatics/btaf108

**Published:** 2025-03-14

**Authors:** Brittany Lasher, David A Hendrix

**Affiliations:** Department of Biochemistry and Biophysics, Oregon State University, 2011 Agricultural and Life Sciences, 2750 SW Campus Way, Corvallis, Oregon 97331, USA; Department of Biochemistry and Biophysics, Oregon State University, 2011 Agricultural and Life Sciences, 2750 SW Campus Way, Corvallis, Oregon 97331, USA; School of Electrical Engineering and Computer Science, Oregon State University, Kelley Egineering Center, 1148, 2461 SW Campus Way, Corvallis, Oregon 97331, USA

## Abstract

**Motivation:**

RNA secondary structure is often essential to function. Recent work has led to the development of high-throughput experimental probing methods for structure determination. Although structure is more conserved than primary sequence, much of the bioinformatics pipelines to connect RNA structure to function rely on nucleotide sequence alignments rather than structural similarity. There is a need to develop methods for secondary structure comparisons that are also fast and efficient to navigate the vast amounts of structural data. K-mer based similarity approaches are valued for their computational efficiency and have been applied for protein, DNA, and RNA primary sequences. However, these approaches have yet to be implemented for RNA secondary structure.

**Results:**

Our method, bpRNA-CosMoS, fills this gap by using k-mers and length-weighted cosine similarity to compute similarity scores between RNA structures. bpRNA-CosMoS is built upon the bpRNA structure array, which represents the structural category of each nucleotide as a single-character structural code (e.g. hairpin=H, etc.). A structural comparison score is calculated through cosine similarity of the k-mer count vectors, generated from structure arrays. A major challenge with k-mer based methods is that they often ignore the length of the sequences being compared. We have overcome this with a length-weighted penalty that addresses cases of two RNAs of vastly different lengths. In addition, the use of “fuzzy counting” has added some optional flexibility to decrease the negative impact that small structural variations have on the similarity score. This results in a robust and efficient way to identify structural comparisons across large datasets.

**Availability and implementation:**

The code and application guidelines of bpRNA-CosMoS are made available at github (https://github.com/BLasher113/bpRNA-CosMoS) and Zenodo (10.5281/zenodo.14715285).

## 1 Introduction

RNA structure is more conserved than primary sequence (Biesiada *et al.* 2022), which makes understanding and analyzing structure key to determining function. Recent progress has led to high-throughput experimental-based approaches for generating RNA secondary structure through chemical probing methods (Kudla *et al.* 2011, Lucks *et al.* 2011, Ding *et al.* 2014, Siegfried *et al.* 2014, Silverman *et al.* 2014, Talkish *et al.* 2014, Spitale *et al.* 2015, Sugimoto *et al.* 2015, Ghut *et al.* 2016, Lu *et al.* 2016, Nguyen *et al.* 2016, Sharma *et al.* 2016, Ritchey *et al.* 2017, Metkar *et al.* 2018, Ziv *et al.* 2018, Morf *et al.* 2019a, Zinshteyn *et al.* 2019, Cai *et al.* 2020, Twittenhoff *et al.* 2020, Weng *et al.* 2020, Lajarte *et al.* 2024) resulting in high accuracy structural predictions. With these advances comes the emergence of an enormous quantity of secondary structure data to be studied and categorized (He *et al.* 2024, Li *et al.* 2021, Lajarte *et al.* 2024). This emphasizes the need to develop methods to compare and classify secondary structures in a fast and efficient manner, enabling assignments of functional annotation through the connection between structure and function.

Although methods to compare RNA secondary structure exist (Höchsmann *et al.* 2003, Mattei *et al.* 2015, Lasher and Hendrix 2023, Wang *et al.* 2023), these approaches are often not designed for immense amounts of data. A key challenge remaining within the RNA comparison field is to decrease the time-complexity to provide a quick and effective way to calculate similarity between RNA structures. Many of the existing approaches involve alignment-based strategies that rely on dynamic programing or tree-based approaches, both of which result in a higher time-complexity of $O(L_{1}L_{2})$ or greater (Höchsmann *et al.* 2003, Mattei *et al.* 2015, Chao *et al.* 2022, Lasher and Hendrix 2023, Wang *et al.* 2023), being impractical to apply on large datasets. In our previous work, we developed bpRNA-align (Lasher and Hendrix 2023), a customized global RNA structure alignment approach, applying an inverted context-specific affine gap penalty and a feature-specific substitution matrix. Other similar alignment-based approaches that use dynamic programing include BEAGLE (Mattei *et al.* 2015) and RNAsmc (Wang *et al.* 2023). RNAforester is yet another type of approach that also produces an output alignment but uses a tree-based strategy (Höchsmann *et al.* 2003). While these methods provide a detailed comparison on the nucleotide-level through output alignments, this is not always necessary for determining similarity between structures and providing functional classifications. A successful alignment-free approach, super-n-motifs, uses a bag-of-n-motifs model of secondary structure, generates a reduced set of motifs based on relative importance, and applies singular value decomposition to generate a vector-based representation (Glouzon *et al.* 2017).

Alignment-free methods are promising for their fast and effective results. k-mer similarity approaches have shown success as alignment-free strategies and are already valued for being fast and accurate when applied to protein sequence (Mirdita *et al.* 2019, Chang *et al.* 2023), and DNA/RNA sequence (Bray *et al.* 2016, Bussi *et al.* 2021, Teng *et al.* 2023, Uddin *et al.* 2023). For example, a recent successful study has applied nucleotide sequence k-mer profiles and Jaccard similarity to measure functional similarity between lncRNAs (Teng *et al.* 2023). Other results have demonstrated that high accuracy and low time and space complexity can be achieved for DNA sequence similarity utilizing a k-mer based approach (Uddin *et al.* 2023). Within protein sequence, the Snekmer software has been developed to perform clustering for determination of protein families by representing proteins as amino acid k-mer vectors. This has allowed for links between sequences of distant sequence similarity, resulting in accurate classification of challenging protein families (Chang *et al.* 2023). These studies are numerous, highlighting the success of k-mer based approaches within various biological fields. However, they have yet to be implemented on RNA secondary structure, which may be in part because secondary structure is 2D and that may not be considered as compatible with k-mers. While sequence representations of both protein and RNA secondary structures exist, RNA structure representations such as the dot bracket notation (DBN), do not differentiate between loop types, which limits the utility of a k-mer approach. However, other representations provide more structural information by incorporating detailed loop-types (Danaee *et al.* 2018). bpRNA, an automated structure annotation method developed in our prior work generates an RNA feature-dense structure representation known as the “structure array” (Danaee *et al.* 2018), which is similar to the Washington University Secondary Structure (WUSS) notation, but provides distinction between multiloops and external loops (Danaee *et al.* 2018), and is used in the generation of the bpRNA-1m (https://bprna.cgrb.oregonstate.edu) database containing over 100 000 annotated RNAs from 7 different source databases (Danaee *et al.* 2018). The structure array specifies stem and loop type information on the nucleotide-level through the following characters: H (hairpin), M (multiloop), X (external loop), I (internal loop), B (bulge), E (end), L (left-handed stem), and R (right-handed stem). The structure array characters offer an intuitive language to represent RNA structures and provides a compact loop-specific input for secondary structure comparison approaches, such as k-mer based methods.

**Figure 1.**
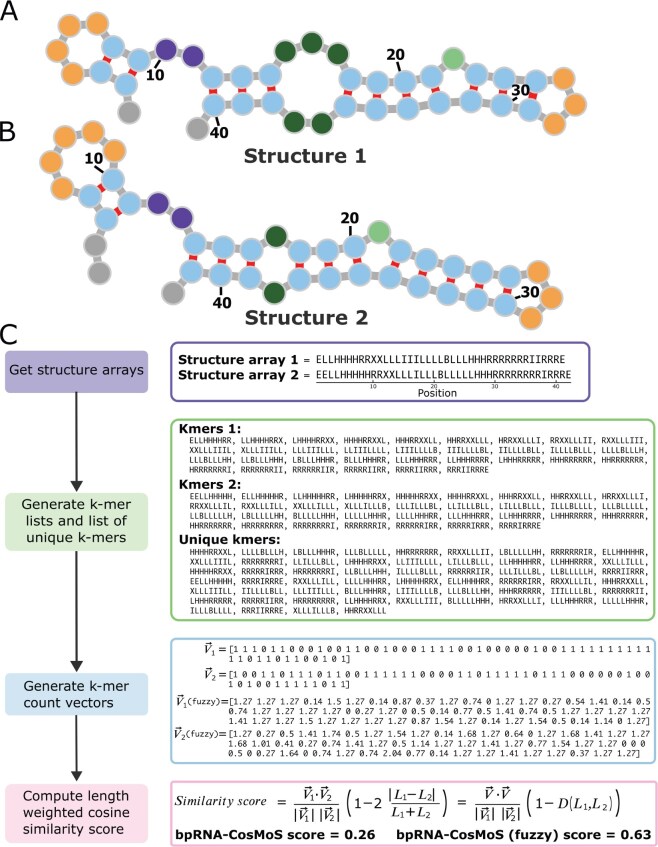
bpRNA-CosMoS example comparison case. (A and B) RNA examples with a high level of structural similarity to use for comparison with bpRNA-CosMoS. (C) bpRNACosMoS flowchart example.

We present bpRNA-CosMoS, an efficient and accurate method using k-mer-based cosine measure of similarity (CosMoS) applied to k-mer count vectors from the bpRNA structure array, for computing RNA structural similarity (Fig. 1). A key challenge of this approach is addressing length differences between RNAs being compared, which can sometimes result in an abnormally high similarity score. We overcome this through the enactment of a length-weighted penalty, which addresses cases where two RNAs are of vastly different lengths and have a common overpowering structural element, resulting in k-mer count vectors that point in a similar direction. In addition, bpRNA-CosMoS provides optional flexibility through “fuzzy counting”, which decreases the negative impact that small structural variations have on the comparison score. This results in a low time-complexity approach for identifying structural comparisons across vast amounts of data.

Enabling structural comparisons on a large-scale will have a significant impact to the bioinformatics field by providing the foundation for further research into identification and analysis of structural classifications. This will facilitate the generation of RNA train and test datasets for machine learning composed of low structural overlap between test and train data. RNA datasets have previously been generated with limited sequence identity between train and test (Rivas *et al.* 2012), but this has not been done on the structural level. Furthermore, these structural classifications can be used to better understand the connection between structure and function, resolving the extent to which structure is required for the binding events between noncoding RNAs and RNA binding proteins (RBPs) (Dominguez *et al.* 2018).

## 2 Materials and methods

### 2.1 Computing cosine similarity scores

Two RNAs were compared (Fig. 1A and B) by first generating their corresponding k-mer lists from the bpRNA structure arrays (Fig. 1C). The unique set of k-mers was used along with the k-mer lists to compute the count vectors, $\vec{V_{r}}$, for each RNA, r. To evaluate the level of similarity, we calculated the cosine similarity score between k-mer count vectors, $\vec{V_{r}}$, by $\cos\theta ={\vec{V}}_{1}\cdot {\vec{V}}_{2}/\left|\vec{V_{1}}\right|\left|\vec{V_{2}}\right|$.

### 2.2 Unique k-mers and edit distance information

**Figure 2.**
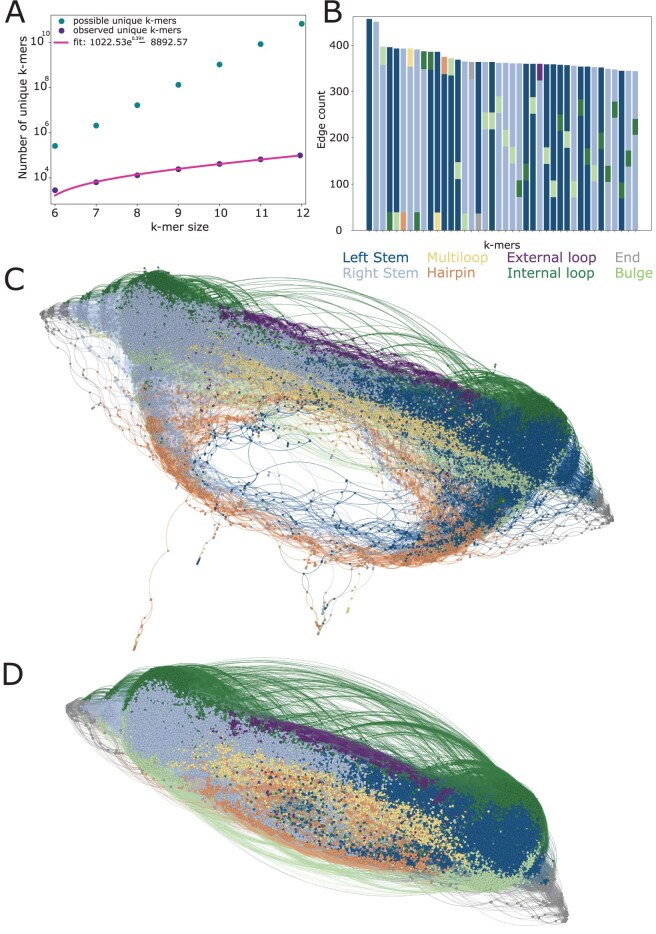
Visualization of the k-mer space. (A) The number of possible and observed unique k-mers within the bpRNA database over a range of k-mer sizes. (B) The top 20 kmers participating in the highest number of connections. (C) Network plot of the k-mer space for edges of edit distance ≤ 1. (D) Network plot of the k-mer space for edges of edit distance ≤ 2.

To describe the space of k-mers under consideration and the similarity between k-mers, we first detected the set of unique k-mers within the bpRNA-1m meta-database (Fig. 2A), containing over 100 000 structures (Danaee *et al.* 2018). Next, bpRNA-align was applied to generate an alignment for each unique pair of k-mers. The optimal alignment yields the edit-distance, d, by counting the number of insertions, deletions, or mismatches. This enables us to determine the level of fuzziness to add between k-mers through pseudo counts for the bpRNA-CosMoS (fuzzy) approach. To do so, we examined two levels of fuzziness, with the less fuzzy option having pseudo counts added for ≤1, and the more fuzzy option having pseudo counts added for d≤2.

### 2.3 Visualization of k-mer space

The k-mer space is composed of all unique k-mers observed within the bpRNA-1m database, where edges exist between k-mer pairs only for d≤1 or 2. Figure 2B shows the top k-mers based on the number of edge connections. To visualize the k-mer space, the node and edge data was viewed within Gephi (Bastian *et al.* 2009), a network visualization software, with node colors representing the structural elements with the highest occurrence in each k-mer (e.g., LLLLLLHHH is labeled based on the color corresponding to L) (Fig. 2C and D). This shows the comparison between different possible k-mers and is used to determine the impact of an edit distance of 1 or 2, guiding the bpRNA-CosMoS (fuzzy) selection of the level of fuzziness. Furthermore, any RNA structure can be represented as a subset of vertices within the multigraph that represents the k-mer space visualized in Fig. 2.

### 2.4 Length weighting

To address comparing RNA structures with different lengths, which may result in count vectors pointing in a similar direction, we applied an RNA length penalty. For two sequences of length $L_{1}$ and $L_{2}$, the penalty, $D(L_{1},L_{2})$, chosen was the absolute length difference divided by the average length:

(1) $$D(L_{1},L_{2})=\frac{2\left|L_{1}-L_{2}\right|}{L_{1}+L_{2}}$$

This penalty term can then be used to reduce the cosine similarity scores for RNAs with different lengths according to:

(2) $$S(V_{1},V_{2})=\frac{{\vec{V}}_{1}\cdot {\vec{V}}_{2}}{\left|{\vec{V}}_{1}\right|\left|{\vec{V}}_{2}\right|}\left(1-D\left(L_{1},L_{2}\right)\right)$$

**Figure 3.**
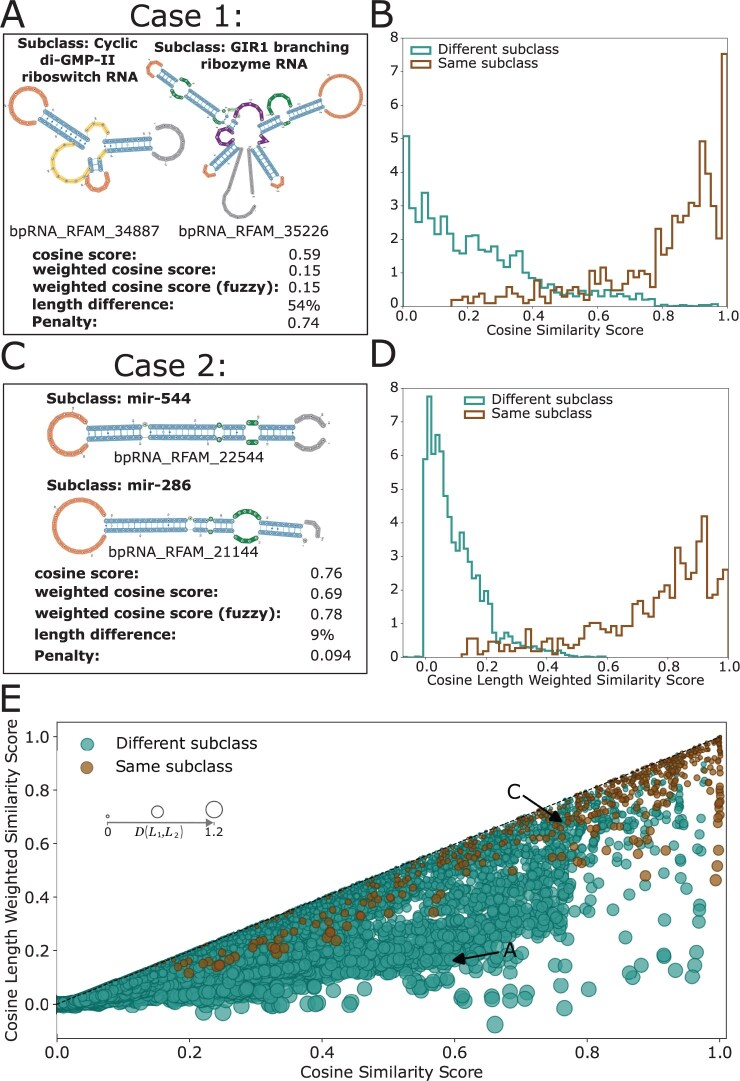
Implementation and application of cosine similarity scores and the length weighted approach on the benchmark dataset. (A) Example case 1, a comparison of varied length RNAs from different RNA subfamilies. (B) Cosine similarity score distribution for RNA pairs form the same subclass and different RNA subclasses with length differences < 0.30. (C) Example case 2, a comparison of similar length RNAs from different RNA subfamilies. (D) Cosine similarity score distribution for RNA pairs with differences < 0.30. (E) Cosine length weighted similarity scores versus cosine similarity scores for RNA pairs from the same RNA subfamilies and from different subfamilies, with the size of points based on the score penalty.

To evaluate our length weighted approach, a scatter plot was generated between nonweighted and weighted scores, in addition to score distribution plots (Fig. 3B, D, and E). For the score distributions, RNA pairs from different classes with a length difference <30% were excluded to examine pairs with larger length variation.

### 2.5 Optional bpRNA-CosMoS (fuzzy) approach

We developed bpRNA-CosMoS (fuzzy) to handle cases where minor structural variability causes unnaturally low scores. This is a result of the k-mer based approach, where even a single nucleotide structural change can produce distinct sets of k-mers, leading to inaccurate scores. To correct for this, the fuzzy option adds pseudo-counts for k-mers that are closely related, having an edit distance ≤ 2 between them. Fractional pseudo-counts are added to the count vectors for k-mers an edit distance of d away by:

(3) $$\mathrm{pseudo}\_\mathrm{count}=e^{-d}$$

The specific formula is not important, but used to decrease as a function of the distance from the contributing k-mer.

### 2.6 Optimizing k-mer size for bpRNA structure arrays

**Figure 4.**
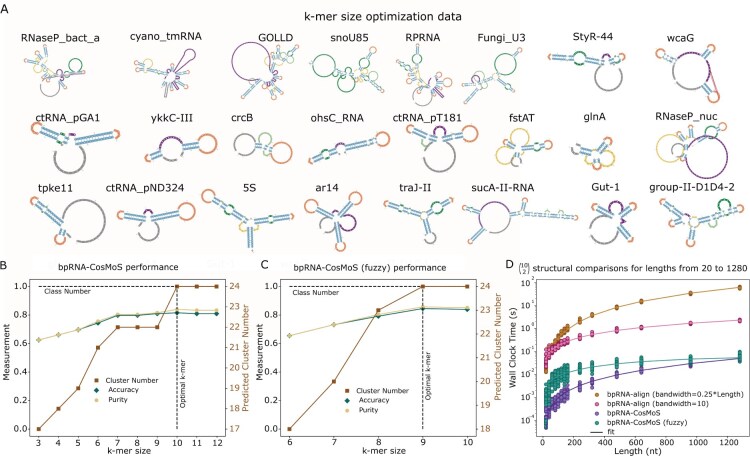
Optimization of bpRNA-CosMoS. (A) k-mer size optimization dataset containing 24 subclasses. (B) bpRNA-CosMoS optimization of k-mer size through analysis of purity, accuracy, and cluster number versus varied k-mer sizes. (C) bpRNA-CosMoS (fuzzy) optimization of k-mer size through analysis of purity, accuracy, and cluster number versus varied k-mer sizes. (D) Plot of log-scale wall clock time versus length over a range of lengths from 20 to 1280 nucleotides.

To optimize the k-mer size, a dataset consisting of 7 randomly chosen structures for 24 different RNA subclasses was collated from the bpRNA-1m meta database (A). These subclasses were excluded from use in the future method comparison and example cases. Similarity scores were calculated using both bpRNA-CosMoS and bpRNA-CosMoS (fuzzy), with a ranged set of k-mer sizes from 3–12 and 6–10, respectively. The reason for stopping bpRNA-CosMoS (fuzzy) at *k* = 10 is because the performance plateaued and the time complexity is prohibitive for *k* > 10. True labels were known based on the RNA subclasses, and cluster predictions were determined using affinity propagation clustering, which is an ideal approach because it does not require a set number of clusters, it takes affinity or similarity scores directly, and it does not involve setting an edge score threshold. The performance of purity, accuracy, and cluster number metrics were used to select the optimal k-mer size, k (Fig. 4B and C).

### 2.7 Wall clock time comparison

To compare the wall-clock time of bpRNA-CosMoS to bpRNA-CosMoS (fuzzy), and bpRNA-align, a dataset with a broad range of lengths, from 20 to 1280, was compiled from the bpRNA-1m meta database. For each length, 10 RNAs were randomly chosen with an allowed length variation of 10%. The wall clock time for each method was determined as the average time to compute sampled RNA pairs, using 45 comparisons for each length category (Fig. 4D).

### 2.8 Generation of the test datasets

**Figure 5.**
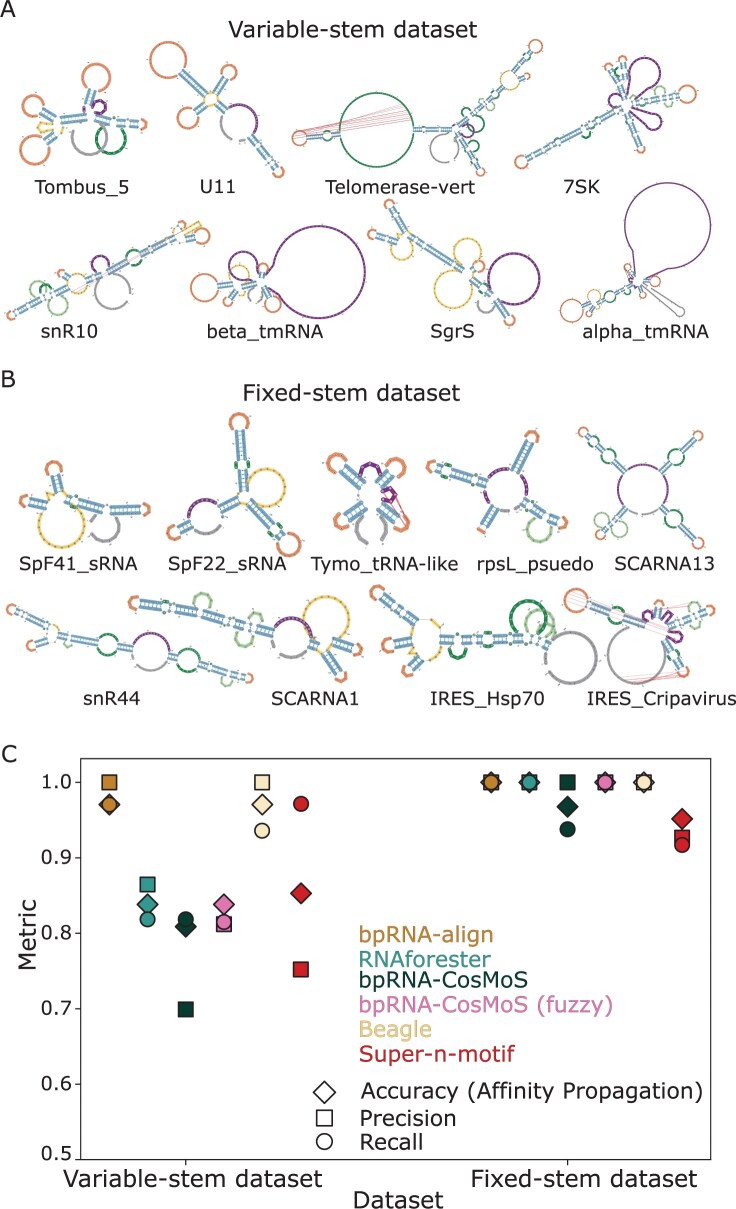
Test datasets and comparison of bpRNA-CosMoS to alignment, tree, and alignment free based methods. (A) Variable-stem dataset composition. (B) Fixed-stem dataset composition. (C) Comparison of bpRNA-CosMoS to bpRNA-align (bandwidth of 25% of the length), RNAforester, Beagle, and Super-n-motifs through metrics of accuracy, precision, and recall.

To compare bpRNA-CosMoS to other methods, we generated the variable-stem and fixed-stem datasets. For each dataset, structures were chosen from the bpRNA-1m meta-database (Danaee *et al.* 2018) and were selected to have a reduced redundancy by meeting a 90% or less sequence similarity criterion. The variable-stem dataset is composed of 8 different RNA subclasses, with each subclass containing between 4 and 12 unique RNAs with a variable number of stems both within and across subclasses (Fig. 5A). This test set is designed to have a larger intra-subclass length variation (Table 1), posing a challenge for all methods. The fixed-stem dataset is composed of nine different RNA subclasses, with each subclass containing between four and nine RNAs (Fig. 5B). This dataset was chosen to have a fixed number of stems (four) with a large inter-subclass length variation (Table 1). This is designed to provide a more challenging test set for the alignment-free methods, which do not directly account of RNA length. The observed changes in the number of RNAs per each subclass is due to the number of available RNAs that also meet the reduced redundancy criterion.

**Table 1.** Metrics analyzed for each RNA subclass from the bpRNA-1m meta database chosen in the selection of the variable-stem and fixed-stem datasets.

RNA subtype	RNA count	Avg Segment	Length std	Avg Length
Variable-stem dataset				
alpha_tmRNA	11	6	68.3	376.82
SgrS	8	5.37	12.19	230.375
beta_tmRNA	6	5.7	49.48	302.67
snR10	12	5.83	13.17	247.58
7SK	4	6	11.38	313.75
Telomerase-vert	10	5	29.89	447.1
U11	10	5	12.26	139.9
Tombus_5	7	5	14.49	159.14
Fixed-stem dataset				
snR44	4	4	12.89	203.25
SpF41_sRNA	9	4	2.16	105.56
SpF22_sRNA	5	4	6.25	142.6
Tymo_tRNA-like	8	4	2.47	82.86
SCARNA1	7	4	2.49	164.29
SCARNA13	8	4	5.23	273.86
rpsL_pseudo	8	4	7.29	127.25
IRES_Cripavirus	7	4	4.92	198.7
IRES_Hsp70	6	4	12.95	206.83

### 2.9 Comparison of bpRNA-CosMoS to other methods

To compare the results of bpRNA-CosMoS to bpRNA-align (Lasher and Hendrix 2023), RNAforester (Höchsmann *et al.* 2003), Beagle (Mattei *et al.* 2015), and super-n-motifs (Glouzon *et al.* 2017) the fixed-stem and variable-stem test datasets were used. Similarity scores from the bpRNA-CosMoS methods were generated for all unique pairs within the dataset. Affinity propagation clustering was applied to gain cluster predictions and accuracy, precision and recall metrics were computed to evaluate performance (Fig. 5C).

With the time complexity of bpRNA-CosMoS being $O\left(L-k+1\right)$, which is lower than the 25% bandwidth applied for bpRNA-align$,\;O\left(L^{2}/4\right)$, we aimed to provide a more realistic time complexity comparison between the two approaches by restricting the bpRNA-align bandwidth to be on par with the k-mer length. We implemented a bandwidth of 10 and tested it on both the test datasets and the optimization dataset.

### 2.10 Large clustering examples using bpRNA-CosMoS

**Figure 6.**
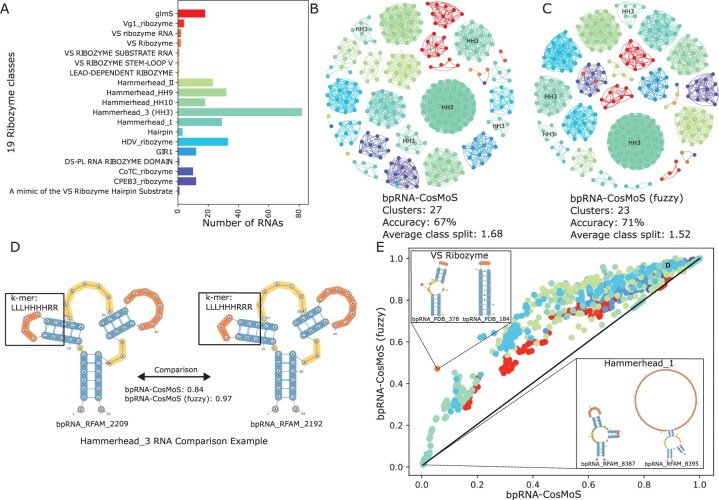
Application of bpRNA-CosMoS and bpRNA-CosMoS scores to cluster Ribozyme RNA biological classes based on structure alone. (A) Bar plot of the ribozyme RNA classes and the number of RNAs in each class. (B and C) bpRNA-CosMoS and bpRNA-CosMoS (fuzzy) clustering results with edges drawn between RNAs of the same cluster and colored by true RNA class. (D) Example comparison between two RNAs from the same RNA class, where the bpRNA-CosMoS clustering approach predicts them to be in different clusters, but the bpRNA-CosMoS (fuzzy) clustering approach predicts them to be in the same cluster. (E) Comparison of bpRNA-CosMoS and bpRNA-CosMoS (fuzzy) scores for the RNA pairs of the same class and colored by the RNA class.

**Figure 7.**
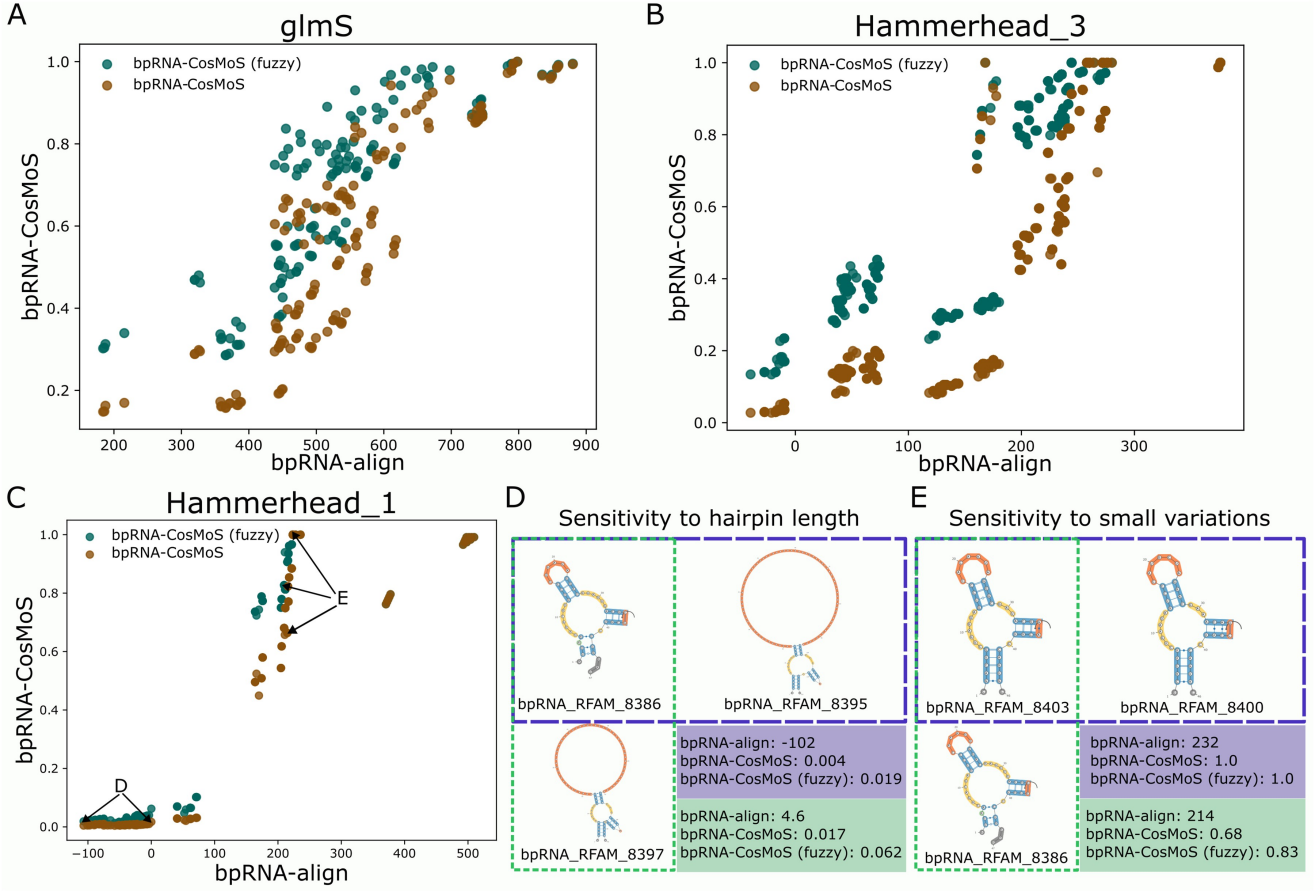
Examination of bpRNA-CosMoS scores to bpRNA-align scores for same subclasses within the Ribozyme RNA class. (A) bpRNA-CosMoS versus bpRNA-align score comparison for the glmS RNA subclass. (B) bpRNA-CosMoS versus bpRNA-align score comparison for the Hammerhead_3 RNA subclass (C) bpRNA-CosMoS versus bpRNA-align score comparison for the Hammerhead_1 RNA subclass. (D) Score comparison between two different pairs of RNAs, where bpRNA-align shows a large separation and bpRNA-CosMoS shows very little separation. (E) Score comparison between two different pairs of RNAs, where bpRNA-CosMoS shows a large separation and bpRNA-align shows very little separation.

To provide use-case examples for bpRNA-CosMoS, we applied it on both the ribozyme RNA class and the internal ribosome entry site (IRES) RNA class from the bpRNA-1m meta-database. The number of subclasses in the ribozyme and the IRES datasets are 19 and 33, respectively, with each subclass containing structure counts ranging from 1–82 to 1–92 (Fig. 6A, Supplementary Fig. S3A). Subclasses were distinctly colored within the bar and network plots (Fig. 6A–C) and scores were clustered using affinity propagation clustering, with edges representing the connections between structures within a cluster. To identify differences between bpRNA-CosMoS and bpRNA-CosMoS (fuzzy), a scatter plot of the scores was generated for structural pairs from the same RNA subclass (Fig. 6E). Furthermore, to understand the correlation between scores from bpRNA-align and bpRNA-CosMoS, we examined single RNA subclass scatter plots within the Ribozyme RNA class (Fig. 7).

## 3 Results

### 3.1 Computing cosine similarity scores

When calculating cosine similarity between pairs of structures, it was observed that a high similarity score could exist between two distinct RNA structures that had varied lengths. For example, case 1 (Fig. 3A) shows visually unique structures, originating from different RNA classes with a length difference of 54%. The resulting score between the pair of 0.59, indicates a higher level of similarity than expected from visual observation. Further exploration into the cause revealed that the count vectors pointed in a similar direction due to the population of certain overpowering k-mers. This effect is seen in the score distribution plot for the benchmark dataset, where there is an overlap in scores between pairs of structures from the same subclass and from different subclasses (Fig. 3B). In general, structures from the same subclass will have higher similarity than structures from different subclasses. Due to the challenges in scoring with length differences, this trend was not fully observed (Fig. 3B), as scores from different subclasses reached up to ≈0.98.

There are cases of RNAs from different subclasses with similar lengths that have high similarity scores and present visibly similar structures (Fig. 3C). Unfortunately, k-mer based similarity scores do not always capture these types of small structural differences that separate RNAs into different subclasses. For example, case 2 shows the comparison of miRNA structures from different subclasses (Fig. 3C), which have little length variation. Even with minute changes in the positioning of internal loops and bulges between the structures, a high similarity score is still obtained.

### 3.2 Length weighting cosine similarity scores

To correct for length differences leading to unexpectantly high scores, a length penalty was developed. Although other common scoring approaches were attempted, including the Pearson correlation coefficient (Vinga and Almeida 2003, Vinga 2007), Euclidean distance (Vinga 2007), and Euclidean similarity (Supplementary Fig. S1), they were not effective due to the inherent nature of RNA secondary structure. Examination of case 1 (Fig. 3A) demonstrates that our length weighted approach can lower the score of two distinct structures, originating from different subclasses (lowered from 0.59 to 0.15). Inspection of the score distribution for this approach resulted in a larger separation in scores between pairs of RNAs from the same subclass and from different subclasses (Fig. 3D); this was not achieved with other scoring approaches (Supplementary Fig. S1). When applied to RNAs from different subclasses with small to no length difference, such as case 2 (Fig. 3C), the length weighted method did not have a big effect, with the score only changing from 0.76 to 0.69.

Comparison of the length weighted similarity score to the cosine similarity score (Fig. 3E) shows larger penalties for RNA pairs from different subclasses, indicating larger length variation. We see a trend for larger penalty cases, where the cosine similarity score is higher than the weighted score, demonstrating the ability of the weighted approach to correct for unexpected high scores due to length variation.

### 3.3 Unique k-mers and edit distance information

For a given k-mer size, the number of unique k-mers observed within the bpRNA-1m meta database is less than the number of possible unique k-mers (Fig. 2A). For example, the k-mer size of 10 has $8^{10}$ ($A^{k}$ for alphabet size A) possible unique k-mers, whereas only 41 555 unique k-mers were observed (Fig. 2A). The edit distance was calculated for each unique pair of k-mers, resulting in approximately 863 million calculations. k-mers with the highest number of edges (edit distance ≤ 2) between other k-mers were mainly composed stems, L or R (Fig. 2B).

### 3.4 Visualization of k-mer space

To observe the effects of fuzziness added with bpRNA-CosMoS (fuzzy) and to determine the desired level of flexibility (d≤2 or d≤1), we generated a network plot visualizing the RNA structural space, with k-mers as the vertices, and the d threshold as edges. This visualization is necessary to determine the level of flexibility or fuzziness that will be added into bpRNA-CosMoS (fuzzy). The corresponding networks (Fig. 2C and D) for k=9 showed that end regions (gray) existed on either side of the plot, with left-handed and right-handed stems moving from opposite ends towards the middle of the network. Internal loops and bulges are most prevalent in segments near either the left-handed or right-handed stems, on either side of the network. External loops, multiloops and hairpins existed between left-handed and right-handed stem regions. When looking at the network plot with an edge threshold of an edit distance d≤1, a large gap is seen between hairpin k-mers and other loop types. When the edge threshold is increased to d≤2, the gap previously observed disappears as more edge connections are made. When the edit distance is further increased to d≤3, too many connections are made (∼5 million compared to ∼1 million for d≤2), overly blurring the distinction between categories of k-mers (Supplementary Fig. S2). This unique visualization provides us insight into the organization of the structural k-mers and how the edit-distance threshold will change the scoring by allowing for connections that were not previously detected. Because we are aiming for a level of flexibility that provides k-mer connections that do not lead to obvious disruptions or separations in the k-mer space or blurred connections between feature types within the k-mer space, we determined that an edit-distance of ≤2 provides the optimal fuzziness required to capture similar k-mers within bpRNA-CosMoS (fuzzy).

### 3.5 Optional bpRNA-CosMoS (fuzzy) approach

To address small structural differences that result in unnaturally low scores for structures with visibly high similarity, bpRNA-CosMoS (fuzzy) was created. The addition of pseudo counts within the approach allows for flexibility. For example, Fig. 1A and B shows the comparison between two visibly similar structures. The bpRNA-CosMoS score of 0.26 does not accurately reflect the level of similarity observed. The small differences in structure between these RNAs results in very different k-mer lists. Because of this, the count vectors have many zero count elements (Fig. 1C), and point in different directions, resulting in a score that does not capture the similarity. However, when pseudo counts are added, fewer zero-count elements are observed, and the count vectors point in a similar direction (Fig. 1C). The number of zero elements in the count vectors decreased from 25 to 4 for vector one and 24 to 6 for vector two. This results in a higher score of 0.63, validating that the added flexibility can correct for these inaccurately low scores.

### 3.6 Optimizing k-mer size for bpRNA structure arrays

To determine the ideal k-mer size for bpRNA-CosMoS (with and without fuzzy), we evaluated the accuracy, purity, and cluster number metrics on the optimization dataset (Fig. 4A). For bpRNA-CosMoS, k-mer sizes of 3–12 were evaluated (Fig. 4B) and for fuzzy we used k-mer sizes of 6–10 (Fig. 4C). The reduced range for fuzzy is due to the higher cost of computing the connections for large k. The accuracy and purity of bpRNA-CosMoS trends upwards till it peaks at k=10. The number of clusters also continues to rise to a k-mer size of 10, where it plateaus at 24 clusters predicted, which matches the number of RNA subclasses. Due to the optimal metric performance, k=10 was selected for bpRNA-CosMoS.

For bpRNA-CosMoS (fuzzy), the purity and accuracy continued to increase, reaching the highest values at k=9. The cluster number also increases till k=9, where it plateaued with 24 clusters, the same number as subclasses. The highest metric performance was achieved at k=9, and so it was selected for k.

### 3.7 Wall clock time comparison

To compare the speed between bpRNA-CosMoS, and our previous bpRNA-align method, a plot of the log wall clock time versus RNA length was generated (Fig. 4D). Although the fuzzy option is slightly slower than without, both these methods are substantially faster than bpRNA-align. As RNA lengths are increased, the speed of bpRNA-CosMoS is less effected, whereas bpRNA-align significantly decreases in speed as length rises. This makes bpRNA-CosMoS a more practical option for identifying similarity within large data.

### 3.8 Comparison of bpRNA-CosMoS to other methods

We compared bpRNA-CosMoS with and without the fuzzy option to bpRNA-align, Beagle, RNAforester, and super-n-motifs on the test datasets (Fig. 5C). For bpRNA-align, the bandwidth was set to 25% of the maximum RNA length for each comparison. Metrics of accuracy, precision and recall were evaluated to determine the performance. We observed that bpRNA-CosMoS performed higher with the fuzzy option than without for both test datasets. For the variable stem dataset, the alignment approaches (Beagle and bpRNA-align) performed the highest, reaching accuracy scores of 97% (Fig. 5C). RNAforester, super-n-motifs, and bpRNA-CosMoS performed substantially lower, obtaining accuracies between 80% and 85%. The super-n-motifs alignment-free approach performed slightly above the bpRNA-CosMoS methods for this dataset.

For the fixed-stem dataset, the three nonalignment-free approaches reached 100% accuracy. This was not surprising, as this dataset was designed to challenge the alignment-free methods by providing structures with the same number of stems but having a high inter-subclass length variation. bpRNA-CosMoS performed higher than super-n-motifs for this dataset, with the fuzzy option outperforming both super-n-motifs and the original bpRNA-CosMoS by reaching 100% accuracy, showing it is less effected by the inter-subclass length variation. bpRNA-align reaches the top performance in both test datasets (as expected due to being a more precise global similarity comparison). However, when the bandwidth of bpRNA-align is restricted to 10, providing a more comparable time complexity, the performance decreases for both the optimization and variable-stem datasets. For the optimization dataset, the accuracy decreases from 0.89 to 0.78 (lower than bpRNA-CosMoS methods) and for the variable-stem dataset accuracy decreased from 0.97 to 0.88. This result confirms expectations that the improved performance of bpRNA-align comes at the cost of added time complexity.

### 3.9 Large clustering examples using bpRNA-CosMoS

The ribozyme and IRES RNA classes were used as an example to demonstrate the application of bpRNA-CosMoS (Fig. 6, Supplementary Fig. S3). For the two RNA classes examined, we observed that more clusters were predicted than subclasses (Fig. 6B and C, Supplementary Fig. S3B and C). The clustering network plots demonstrate how some RNA subclasses are split into multiple clusters (i.e. HH3 is split it into five different clusters in the Ribozyme class), quantified by the average subclass split. For the Ribozyme dataset (Fig. 6) bpRNA-CosMoS (fuzzy) performed higher, due to less subclass splitting. An example that leads to possible splitting is the comparison of the Hammerhead_3 subclass pair shown in Fig. 6D. This pair of RNAs has a single nucleotide structural difference between them, which results in a lower score for bpRNA-CosMoS, causing the two structures to be separated into different clusters. However, bpRNA-CosMoS (fuzzy) has a higher score between the two RNAs and was able to cluster them correctly.

However, there are cases where significant structural variations do exist within a single subclass. This is seen for the HCV subclass within the IRES dataset (Supplementary Fig. S3D), the Hammerhead_1 (Supplementary Fig. S4), and the glmS (Supplementary Fig. S5) subclass in the ribozyme RNA class. These subclasses demonstrate cases where many distinct structures groups exist within a single subclass, resulting in class splitting in both bpRNA-CosMoS approaches. In contrast, there are also cases of singleton structures from different RNA subclasses being clustered together. For example, Supplementary Fig. S6 shows a mixed cluster composed of four different RNA subclasses. Visually, these structures are simple stem-loops with little variation between each other (Supplementary Fig. S6C). The result of clustering these together is not an incorrect choice on the algorithms side but may provide evidence that these structures should be categorized together.

Examination of score comparison plots between structure-pairs of the same class yielded on average higher scores for fuzzy in comparison to bpRNA-CosMoS (Fig. 6E, Supplementary Fig. S3D). In these plots we observe an arching curve, where bpRNA-CosMoS (fuzzy) scores are higher along the lower and mid-range but are comparable at the top end of curve. For example, the VS Ribozyme comparison (Fig. 6E) shows two similar structures with small variations, however, the flexibility in bpRNA-CosMoS (fuzzy) allows for a higher score in comparison to bpRNA-CosMoS. Within these plots, we also observe low scoring comparisons indicating little similarity, something we would not expect within the same subclass. However, as previously mentioned, there are subclass cases that are composed of diverse structural categories, which are the cause of these low scores observed with both bpRNA-CosMoS and bpRNA-CosMoS (fuzzy) (Fig. 6E, Supplementary Fig. S3D). For example, the Hammerhead_1 comparison shown in Fig. 6E, has significantly different lengths for the first hairpin loop, which results in the low score observed.

Examination of scoring plots between bpRNA-CosMoS and bpRNA-align (Fig. 7A–C) for single subclasses, show a sigmoid shaped curve. This trend is most exaggerated for the Hammerhead_1 subclass (Fig. 7C); where we observe that at lower scores bpRNA-align is able to provide better separation between comparisons. For example, Fig. 7D shows the comparison of bpRNA_RFAM_8386 to two distinct RNAs.

It is clear from visualizing the structures that one has larger variation in the hairpin loop than the other, however bpRNA-CosMoS is not able to differentiate between the comparisons of these two different pairs (Fig. 7C and D), resulting in similar scores for both. bpRNA-align on the other hand shows a large separation in the scores between these two pairs (Fig. 7C and D). However, when scores are higher and structures are more similar, bpRNA-CosMoS provides more separation compared to bpRNA-align (Fig. 7C). For example, we observe this in a comparison composed of two structurally identical RNAs and one RNA with small variations (Fig. 7E). The results of these two pairs show small differences in scores for bpRNA-align and a larger separation in scores for bpRNA-CosMoS (Fig. 7C and E).

## 4 Discussion

In our work we show that a k-mer-based approach is effective at computing similarity between RNA structures, particularly with a length-weighted correction and added flexibility from “fuzzy” k-mers. It is perhaps surprising that a k-mer based method, which takes pieces out of context of the secondary structure, which is arguably a 2D object, would capture enough detail to effectively compute a similarity score. However, we observe that naturally occurring k-mers work at this task because of constraints due to the structural context that each character appears in. For example, any subsequence of loop characters (e.g. HHH, BBB, III, MMM, or XXX) always resides in-between stem characters (L and/or R), which limit the kinds of “mutations” that a structure might encounter, making k-mers more useful than expected for comparing RNA secondary structure. Furthermore, the k-mer representation covers the secondary structure sequence with a series of overlapping windows that include enough intermediate context to be effective at computing similarity much like it works for DNA and proteins. Our RNA secondary structure representation, the bpRNA structure array, uses an alphabet with a size in between the number of characters in the DNA and protein alphabets. Just like k-mer-based protein sequence similarity methods overcome challenges in detecting homology for proteins with beta-sheets, our method still works for structures containing noncontiguous features because there are enough overlapping intermediate windows.

bpRNA-CosMoS has a time complexity of $O\left(L-k+1\right)$ in comparison to bpRNA-align $O\left(L^{2}\right)$, without a set bandwidth. However, restricting the bandwidth of bpRNA-align provides a closer time complexity comparison between the two methods. By tightly constraining the bandwidth of bpRNA-align, we force the alignment between the two structures to fall closely along the diagonal, which allows for a smaller number of gaps within each sequence in the alignment. This may be acceptable for cases where each RNA class has little structural variation within it. However, when there is an increased intra-class structural variation, this restriction will lead to lower accuracy, as seen when applied to the variable-stem and optimization dataset where bpRNA-align accuracy decreased substantially and even resulted in poorer performance than bpRNA-CosMoS for the optimization dataset. Because bpRNA-CosMoS relies on k-mers it is not forced along the diagonal and thus is less constrained, leading to superior performance in cases with more intra-class variation, while still achieving low time complexity.

A challenge we encountered when developing bpRNA-CosMoS was that using k-mer count vectors can result in scoring inaccuracies, because the lengths of the original sequences being compared were not accounted for. This was an issue when comparing RNAs of vastly different lengths, which both contained a common overpowering repeated structural character (e.g. LLLLLLLLL), resulting in k-mer count vectors pointing in a similar direction (Fig. 3A). These overpowering characters are more common in RNA structure data in comparison to protein sequence or DNA sequence, due to the inherent ordering of structural elements into blocks. Other common vector comparison approaches we attempted (Vinga 2007) were not designed for RNA secondary structure data and were not able to address this challenge (Supplementary Fig. S1), motivating us to develop a scoring approach that is more applicable to RNA secondary structure data. To overcome this challenge, we developed a length-weighted penalty (Equation (1)) using the absolute difference and the length average to weight cosine similarity scores. This resulted in scores that accurately reflected the similarity observed between structures being compared (Fig. 3A) and led to more separation between the score distributions of structures being compared from the same RNA class, versus a different RNA class (Fig. 3D).

Because bpRNA-CosMoS relies on the bpRNA structure array as an input to the method, it does not currently represent pseudoknots or G-quadruplexes. Inclusion of these structural features within the bpRNA structure array could potentially boost the performance of bpRNA-CosMoS. However, this would involve a new set of unique k-mers due to the added structural features, resulting in recalculation of edit distance and the determination of pseudo-count k-mer pairs.

Although bpRNA-CosMoS and super-n-motifs show some similarities as they are both alignment-free methods, bpRNA-CosMoS does not implement dimensionality reduction, allowing it to maintain more of the original structure information. While the super-n-motifs approach does consider G-quadruplexes and pseudoknot information, which may result in higher performance on datasets heavily relying on these key features, it does not take into account length variation or fuzzy representations as bpRNA-CosMoS does. These additions may enable bpRNA-CosMoS to successfully handle inter-subclass length variations, leading to the observed higher performance for the fixed-stem dataset.

In our work, we observed differences in the optimization and performance of bpRNA-CosMoS depending on the fuzzy option. The varied optimal k-mer size between bpRNA-CosMoS with and without fuzzy is likely due to the inherent flexibility within the fuzzy approach, which enables it to reach its highest performance with a slightly lower k-mer size. Furthermore, when it comes overall performance between the two approaches on the optimization dataset and the test datasets, we observe that bpRNA-CosMoS (fuzzy) performs higher. This observed difference in performance is a result of the added flexibility within the fuzzy option, which enables it to capture similarity between RNAs with small structural differences leading to higher overall accuracy. This implies that users would get higher performance when applying the fuzzy option. However, in cases where there is small inter-subclass variation, it may be beneficial to have a less flexible option when comparing RNAs. The exact option to use will vary case-by-case and depend on the user’s dataset.

Our novel approach enabled visualization of RNA structure through the k-mer space with an edit-distance-based network. This showed a strong correlation to the ordering of structural elements within RNA secondary structure. For example, in RNA structure left- and right-handed stems are connected through hairpins, multiloops, or external loops, whereas internal loops and bulges are connected on either side by right-handed stems or left-handed stems. End regions occur at the beginning or end of the sequence and are connected to either a left- or right-handed stem on the connecting side. The structural k-mer space network has nodes orientated in a similar pattern (Fig. 2C and D), with end (gray) nodes on either side of the plot, and with left- and right-handed stems (blue) connecting other loop types. Bulges and internal loops can be found on either side of the network plot, whereas hairpins, multiloops and external loops are observed in the middle, just as in RNA secondary structure. Increasing the threshold from an edit distance of ≤1 to ≤2 provides a demonstration of how fuzziness effects the k-mer space (Fig. 2C and D). As the level of fuzziness increases, the connections within the network plot also increase and the sparseness and gaps that appeared with an edit distance of ≤1 are filled in. This is due to similar k-mers being permitted to form edges, resulting in some overlap or fuzziness.

In the future, we will take advantage of the low time complexity of bpRNA-CosMoS to compute an initial clustering for large RNA structure datasets. This will enable us to perform structural class categorization and possible identification of new subclasses over a broad and structurally diverse set of RNAs and databases. This approach could help group RNA structures from the different source databases and help remove spurious structures that arise from species-specific insertions and deletions for structures computed from comparative sequence analysis approaches. In practice, bpRNA-CosMoS can be used as an initial clustering that reduces the search space and can then be refined with a more fine-grained bpRNA-align method. Improved methods for accurate structural class categorization can be used to generate new machine learning train and test datasets with no structural class overlap, providing a more rigorous evaluation of RNA structure prediction.

Another application of bpRNA-CosMoS is the comparison of RNA motifs for identification of structural categories that may connect structure to function. This method can further be applied to search and identify structural motifs within larger RNAs, such as full-length transcripts. Although this method is a global comparison approach, by breaking up the larger RNA into overlapping windows of a similar size to the motif of interest, the user could apply bpRNA-CosMoS to search vastly large RNAs for specific motifs. Another broad application includes the use of bpRNA-CosMoS to analyze RNA structures of a conformational ensemble. This could potentially identify more sampled conformations within the ensemble, as well as determine which conformations are most distinct through a low similarity score. These applications highlight just a subset of the ways that bpRNA-CosMoS can be applied to quickly and effectively analyze RNA structure in future work.

## Supplementary Material

btaf108_Supplementary_Data

## Data Availability

The data underlying this article are available in Zenodo, at https://zenodo.org/records/14715285.

## References

[btaf108-B1] Bastian M, Heymann S, Jacomy M. Gephi: an open source software for exploring and manipulating networks. *Proc Int AAAI Conf Web Social Media* 2009;3:361–2.

[btaf108-B2] Biesiada M , Hu MY, Williams LD et al rRNA expansion segment 7 in eukaryotes: from signature fold to tentacles. Nucleic Acids Res 2022;50:10717–32. 36200812 10.1093/nar/gkac844 PMC9561286

[btaf108-B3] Bray NL , Pimentel H, Melsted P et al Near-optimal probabilistic RNA-seq quantification. Nat Biotechnol 2016;34:525–7. 27043002 10.1038/nbt.3519

[btaf108-B4] Bussi Y , Kapon R, Reich Z et al Large-scale k-mer-based analysis of the informational properties of genomes, comparative genomics and taxonomy. PLoS One 2021;16:e0258693. 34648558 10.1371/journal.pone.0258693 PMC8516232

[btaf108-B5] Cai Z , Cao C, Ji L et al RIC-seq for global in situ profiling of RNA–RNA spatial interactions. Nature 2020;582:432–7. 32499643 10.1038/s41586-020-2249-1

[btaf108-B6] Chang CH , Nelson WC, Jerger A et al Snekmer: a scalable pipeline for protein sequence fingerprinting based on amino acid recoding. Bioinf Adv 2023;3:vbad005. 10.1093/bioadv/vbad005 PMC9913046 36789294

[btaf108-B7] Chao J , Tang F, Xu L et al Developments in algorithms for sequence alignment: a review. Biomolecules 2022;12:546. 35454135 10.3390/biom12040546 PMC9024764

[btaf108-B8] Danaee P , Rouches M, Wiley M et al BPRNA: large-scale automated annotation and analysis of RNA secondary structure. Nucleic Acids Res 2018;46:5381–94. 29746666 10.1093/nar/gky285 PMC6009582

[btaf108-B9] Ding Y , Tang Y, Kwok CK et al In vivo genome-wide profiling of RNA secondary structure reveals novel regulatory features. Nature 2014;505:696–700. 24270811 10.1038/nature12756

[btaf108-B10] Dominguez D , Freese P, Alexis MS et al Sequence, structure, and context preferences of human RNA binding proteins. Mol Cell 2018;70:854–67.e9. 29883606 10.1016/j.molcel.2018.05.001 PMC6062212

[btaf108-B12] Ghut J et al In vivo mapping of eukaryotic RNA interactomes reveals principles of higher-order organization and regulation molecular cell article In vivo mapping of eukaryotic RNA interactomes reveals principles of higher-order organization and regulation. Mol Cell 2016;62:603–17. 27184079 10.1016/j.molcel.2016.04.028

[btaf108-B13] Glouzon J-PS , Perreault J-P, Wang S et al The super-n-motifs model: a novel alignment-free approach for representing and comparing RNA secondary structures. Bioinformatics 2017;33:1169–78. 28088762 10.1093/bioinformatics/btw773

[btaf108-B14] He S , Huang R, Townley J et al Ribonanza: deep learning of RNA structure through dual crowdsourcing. bioRxiv, 10.1101/2024.02.24.581671, 2024, preprint: not peer reviewed.

[btaf108-B15] Höchsmann M , Toller T, Giegerich R et al Local similarity in RNA secondary structures. In: *Proceedings of the 2003 IEEE Bioinformatics Conference, CSB 2003*. Institute of Electrical and Electronics Engineers Inc. 2003, 159–68. 16452790

[btaf108-B16] Kudla G , Granneman S, Hahn D et al Cross-linking, ligation, and sequencing of hybrids reveals RNA-RNA interactions in yeast. Proc Natl Acad Sci USA 2011;108:10010–5. 21610164 10.1073/pnas.1017386108 PMC3116431

[btaf108-B17] Lajarte AA, des Taillades YJM, Kalicki C et al Diverse database and machine learning model to narrow the generalization gap in RNA structure prediction. bioRxiv, 10.1101/2024.01.24.577093, 2024, preprint: not peer reviewed. PMC12935039 41739924

[btaf108-B18] Lasher B , Hendrix DA. bpRNA-align: improved RNA secondary structure global alignment for comparing and clustering RNA structures. RNA 2023;29:584–95. 36759128 10.1261/rna.079211.122 PMC10159002

[btaf108-B19] Li P , Zhou X, Xu K et al RASP: an atlas of transcriptome-wide RNA secondary structure probing data. Nucleic Acids Res 2021;49:D183–91. 33068412 10.1093/nar/gkaa880 PMC7779053

[btaf108-B20] Lu Z , Zhang QC, Lee B et al RNA duplex map in living cells reveals higher-order transcriptome structure In brief accession numbers GSE74353 Lu et al resource RNA duplex map in living cells reveals higher-order transcriptome structure. Cell 2016;165:1267–79. 27180905 10.1016/j.cell.2016.04.028 PMC5029792

[btaf108-B21] Lucks JB , Mortimer SA, Trapnell C et al Multiplexed RNA structure characterization with selective 2′-hydroxyl acylation analyzed by primer extension sequencing (SHAPE-Seq). Proc Natl Acad Sci USA 2011;108:11063–8. 21642531 10.1073/pnas.1106501108 PMC3131332

[btaf108-B22] Mattei E , Pietrosanto M, Ferrè F et al Web-Beagle: a web server for the alignment of RNA secondary structures. Nucleic Acids Res 2015;43:W493–7. 25977293 10.1093/nar/gkv489 PMC4489221

[btaf108-B23] Metkar M , Ozadam H, Lajoie BR et al Higher-order organization principles of pre-translational mRNPs. Mol Cell 2018;72:715–26.e3. 30415953 10.1016/j.molcel.2018.09.012 PMC6239896

[btaf108-B24] Mirdita M , Steinegger M, Söding J et al MMseqs2 desktop and local web server app for fast, interactive sequence searches. Bioinformatics 2019;35:2856–8. 30615063 10.1093/bioinformatics/bty1057 PMC6691333

[btaf108-B25] Morf J , Wingett SW, Farabella I et al RNA proximity sequencing reveals the spatial organization of the transcriptome in the nucleus. Nat Biotechnol 2019a;37:793–802. 31267103 10.1038/s41587-019-0166-3

[btaf108-B26] Nguyen TC , Cao X, Yu P et al Mapping RNA–RNA interactome and RNA structure in vivo by MARIO. Nat Commun 2016;7:12023–12. 27338251 10.1038/ncomms12023 PMC4931010

[btaf108-B27] Ritchey LE , Su Z, Tang Y et al Structure-seq2: sensitive and accurate genome-wide profiling of RNA structure in vivo. Nucleic Acids Res 2017;45:e135. 28637286 10.1093/nar/gkx533 PMC5737731

[btaf108-B28] Rivas E , Lang R, Eddy SR et al A range of complex probabilistic models for RNA secondary structure prediction that includes the nearest-neighbor model and more. RNA 2012;18:193–212. 22194308 10.1261/rna.030049.111 PMC3264907

[btaf108-B30] Sharma E et al Global mapping of human RNA–RNA interactions. Molecular Cell 2016;62:618–26. 10.1016/j.molcel.2016.04.030 27184080

[btaf108-B31] Siegfried NA , Busan S, Rice GM et al RNA motif discovery by SHAPE and mutational profiling (SHAPE-MaP). Nat Methods 2014;11:959–65. 25028896 10.1038/nmeth.3029 PMC4259394

[btaf108-B32] Silverman IM , Li F, Alexander A et al RNase-mediated protein footprint sequencing reveals protein-binding sites throughout the human transcriptome. Genome Biol 2014;15:R3–16. 24393486 10.1186/gb-2014-15-1-r3 PMC4053792

[btaf108-B33] Spitale RC , Flynn RA, Zhang QC et al Structural imprints in vivo decode RNA regulatory mechanisms. Nature 2015;519:486–90. 25799993 10.1038/nature14263 PMC4376618

[btaf108-B34] Sugimoto Y , Vigilante A, Darbo E et al hiCLIP reveals the in vivo atlas of mRNA secondary structures recognized by Staufen 1. Nature 2015;519:491–4. 25799984 10.1038/nature14280 PMC4376666

[btaf108-B35] Talkish J , May G, Lin Y et al Mod-seq: high-throughput sequencing for chemical probing of RNA structure. RNA 2014;20:713–20. 24664469 10.1261/rna.042218.113 PMC3988572

[btaf108-B36] Teng Z , Shi L, Yu H et al Measuring functional similarity of lncRNAs based on variable K-mer profiles of nucleotide sequences. Methods 2023;212:21–30. 36813016 10.1016/j.ymeth.2023.02.009

[btaf108-B37] Twittenhoff C , Brandenburg VB, Righetti F et al Lead-seq: transcriptome-wide structure probing in vivo using lead(II) ions. Nucleic Acids Res 2020;48:e71. 32463449 10.1093/nar/gkaa404 PMC7337928

[btaf108-B38] Uddin M , Islam MK, Hassan MR et al A fast and efficient algorithm for DNA sequence similarity identification. Complex Intell Syst 2023;9:1265–80. 10.1007/s40747-022-00846-y PMC9395857 36035628

[btaf108-B39] Vinga S. Biological Sequence Analysis by Vector-Valued Functions: Revisiting Alignment-Free Methodologies for DNA and Protein Classification. New York: Nova Science. 2007, 70–105.

[btaf108-B40] Vinga S , Almeida J. Alignment-free sequence comparison-a review. Bioinformatics 2003;19:513–23. 12611807 10.1093/bioinformatics/btg005

[btaf108-B41] Wang H , Lu X, Zheng H et al RNAsmc: a integrated tool for comparing RNA secondary structure and evaluating allosteric effects. Comput Struct Biotechnol J 2023;21:965–73. 36733704 10.1016/j.csbj.2023.01.007 PMC9876829

[btaf108-B42] Weng X , Gong J, Chen Y et al Keth-seq for transcriptome-wide RNA structure mapping. Nat Chem Biol 2020;16:489–92. 32015521 10.1038/s41589-019-0459-3 PMC7182492

[btaf108-B44] Zinshteyn B , Chan D, England W et al Assaying RNA structure with LASER-Seq. Nucleic Acids Res 2019;47:43–55. 30476193 10.1093/nar/gky1172 PMC6326810

[btaf108-B45] Ziv O , Gabryelska MM, Lun ATL et al COMRADES determines in vivo RNA structures and interactions. Nat Methods 2018;15:785–8. 30202058 10.1038/s41592-018-0121-0 PMC6168409

