# Supplementary material for: bpRNA-CosMoS: a robust and efficient RNA structural comparison method using k-mer based cosine similarity
Source: Bioinformatics. 2025 Mar 14;41(4):btaf108. doi: 10.1093/bioinformatics/btaf108 (PMC12017588; doi:10.1093/bioinformatics/btaf108)
Supplement: btaf108_Supplementary_Data [file btaf108_supplementary_data.zip › bpRNA_CosMoS_supplementary_update.docx]

**Supplementary material for**

**bpRNA-CosMoS: A robust and efficient RNA structural comparison method using k-mer based cosine similarity.**

Brittany Lasher^1^, David A Hendrix^1,2^

1 Department of Biochemistry and Biophysics, Oregon State University, Corvallis Oregon

2 School of Electrical Engineering and Computer Science, Oregon State University, Corvallis Oregon


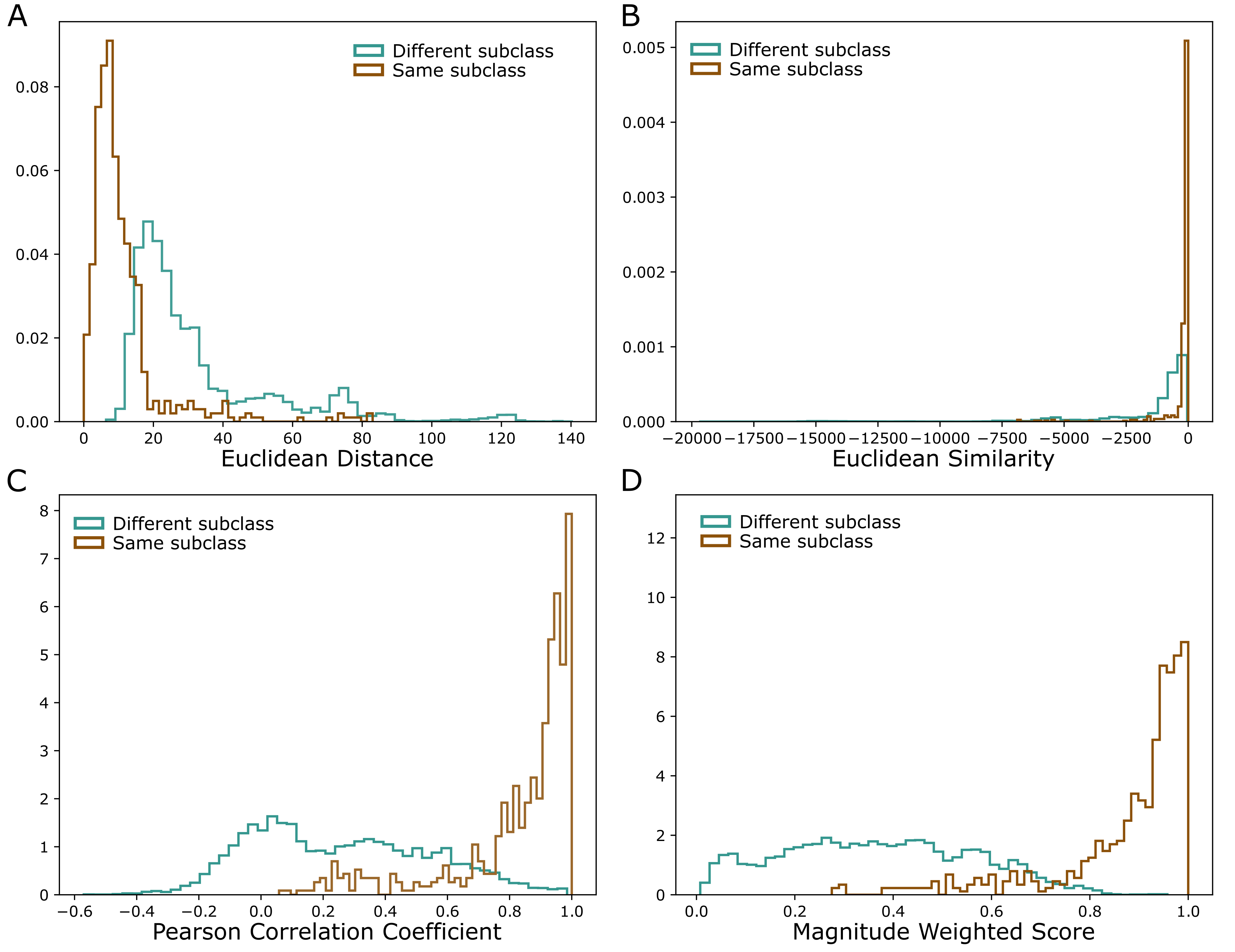
Supplementary Figure 1: Score distributions for different scoring approaches on the benchmark data set. A) Euclidean distance distributions for different subclasses and the same subclasses. B) Euclidean similarity distributions for different subclasses and the same subclasses. C) Pearson correlation coefficient distributions for different subclasses and the same subclasses. D) Magnitude weighted, $\left( \frac{\left| v_{1} \right|-\left| v_{2} \right|}{\left| v_{1} \right|+\left| v_{2} \right|} \right)^{2},$cosine similarity scoring distributions for different subclasses and the same subclasses.


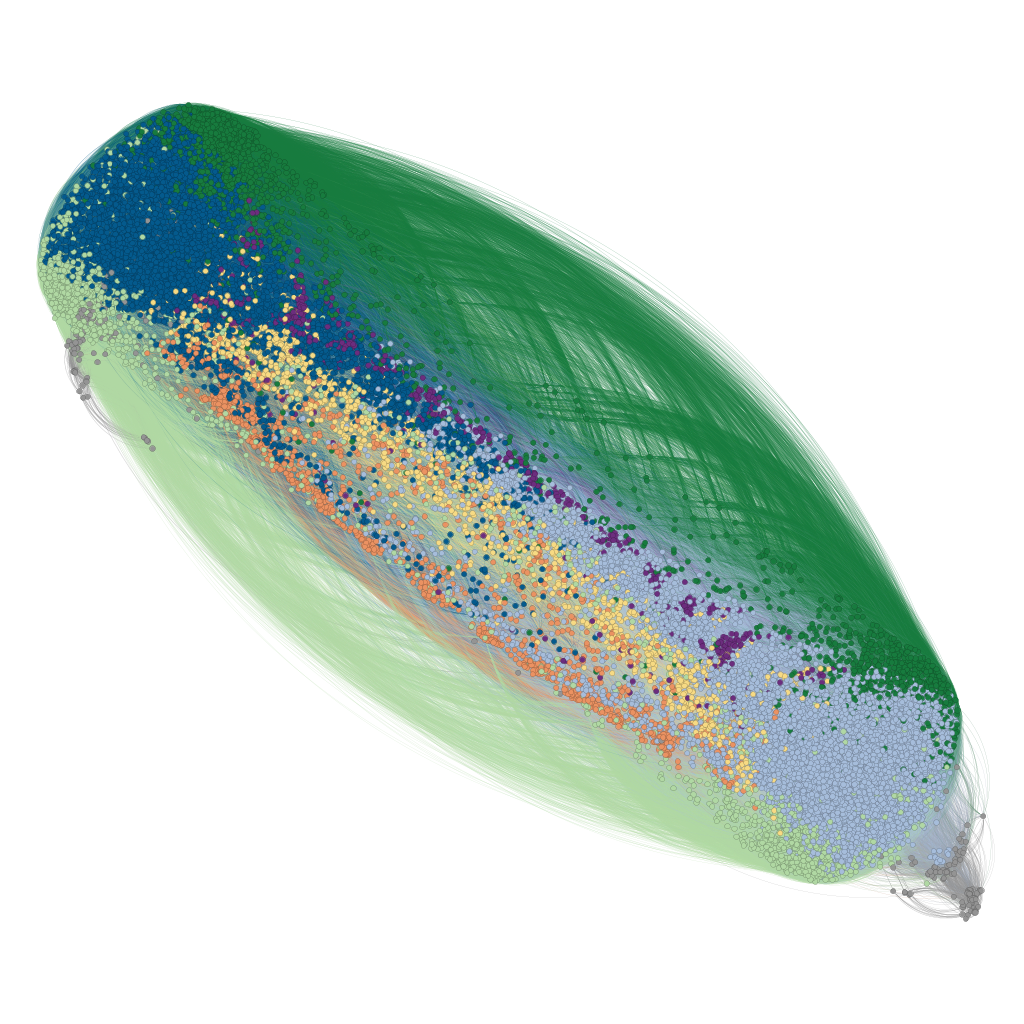
Supplementary Figure 2: Network plot of the k-mer space for edges of edit distance $\leq3$.


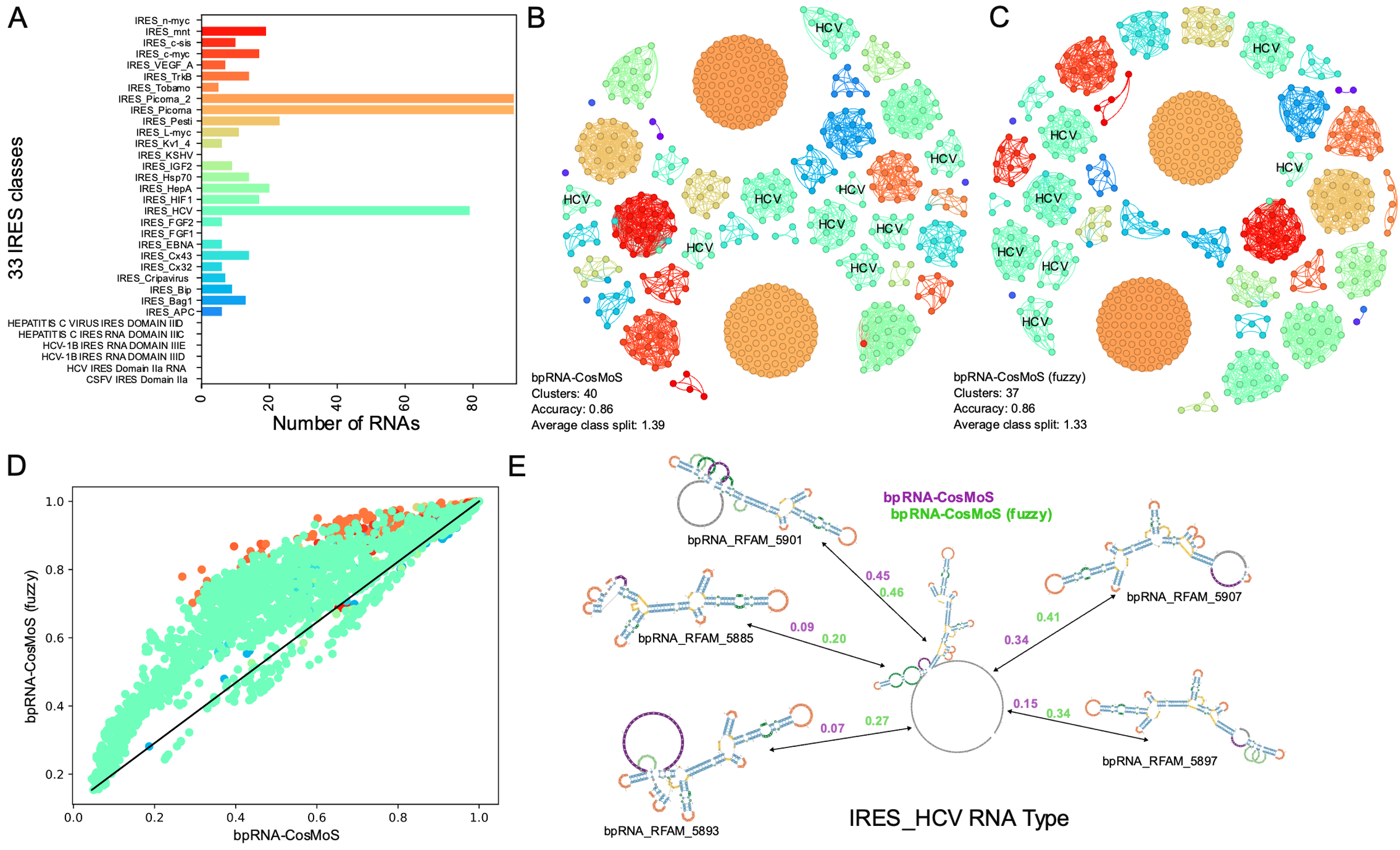
Supplementary Figure 3: Analysis of bpRNA-CosMoS on the IRES RNA subclasses. A) Bar plot of the 33 different IRES subclasses showing the number of RNAs for each. B) bpRNA-CosMoS clustering results. C) bpRNA-CosMoS (fuzzy) clustering results. D) Score comparison between bpRNA-CosMoS approaches. E) Diversity of RNA structural groupings in the HCV RNA subclass.


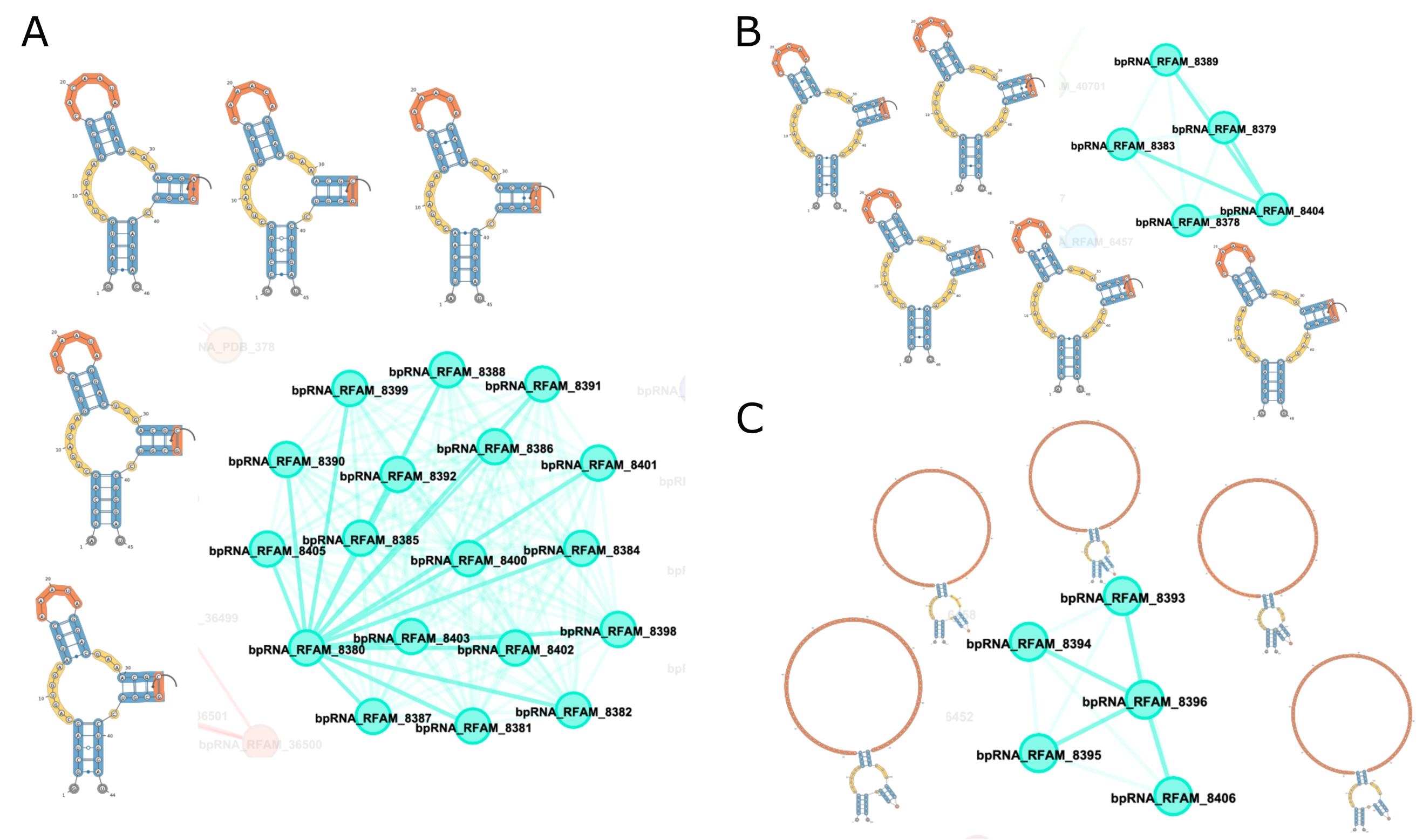
Supplementary Figure 4: Clusters from the Hammerhead_1 Ribozyme RNA subclass. A) Cluster one from the Hammerhead_1 subclass. B) Cluster two from the Hammerhead_2 subclass. C) Cluster three from the Hammerhead_3 subclass.


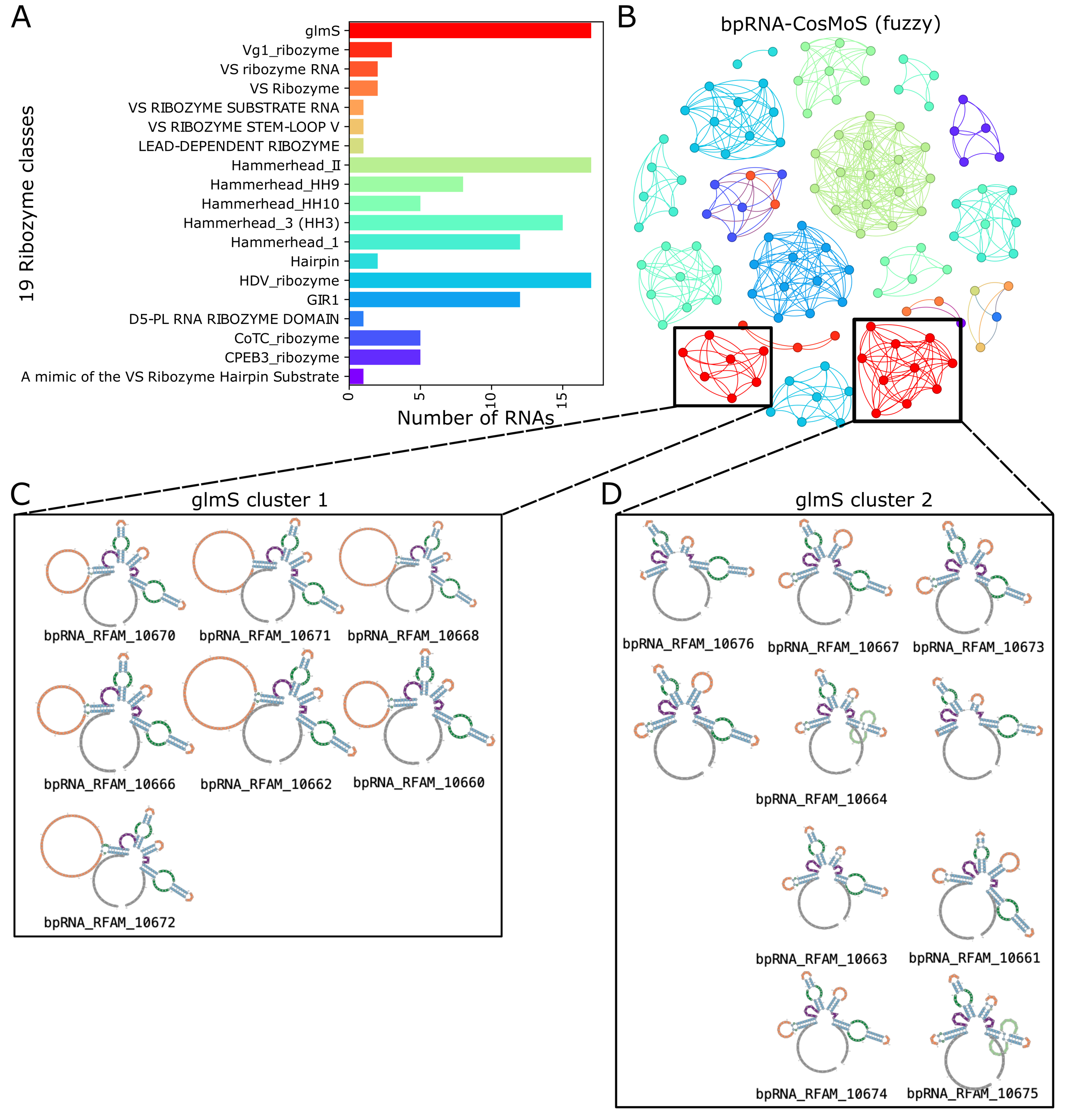
Supplementary Figure 5: Ribozyme clustering splits with the glmS subclass. A) Number of RNAs subclass bar plot. B) Network plot for the Ribozyme RNA class, showing the glmS cluster splits in the boxes. C) glmS cluster one of the Ribozyme subclass. D) glmS cluster two of the Ribozyme subclass.


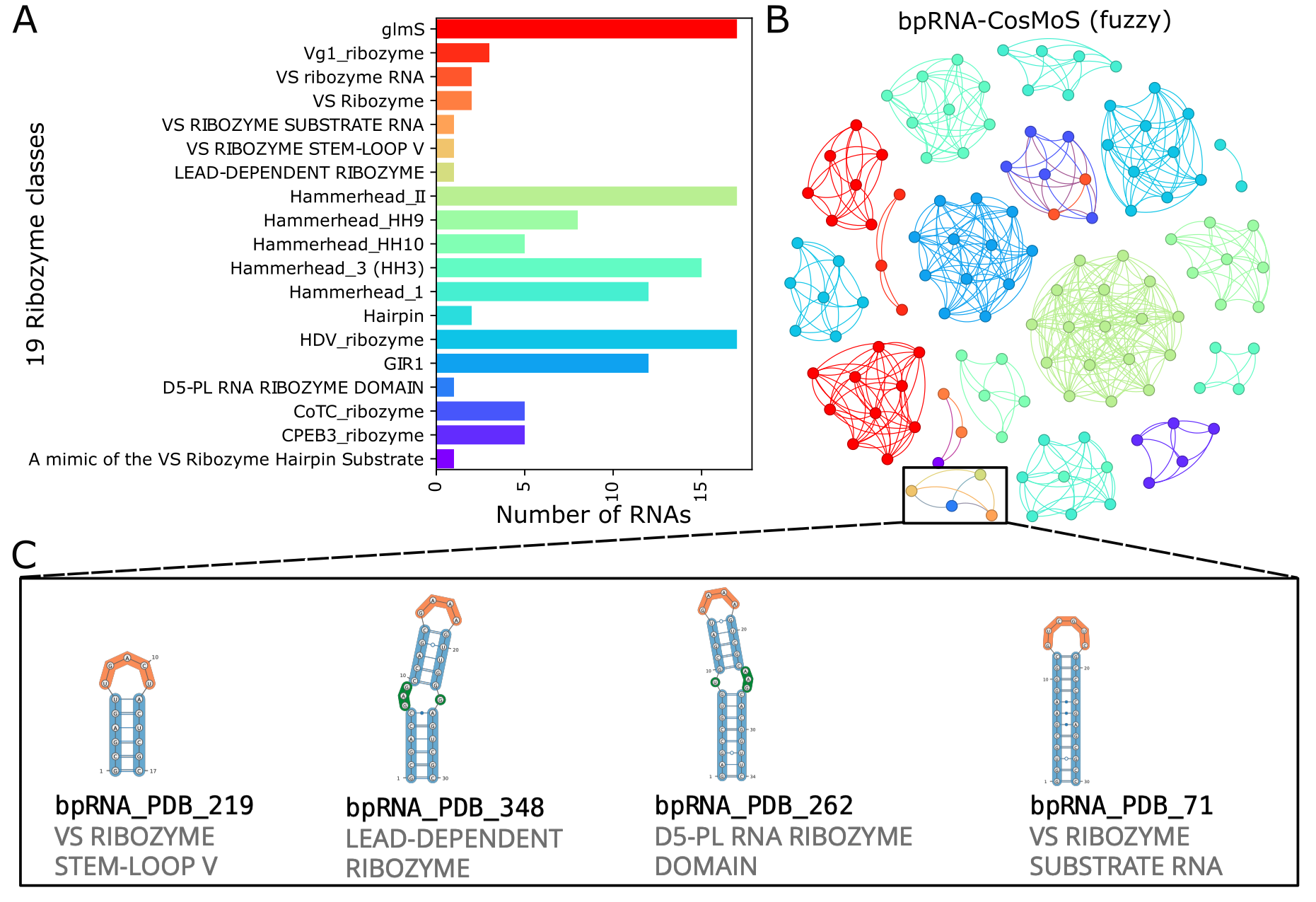
Supplementary Figure 6: Visualization of multi-subclass cluster in the Ribozyme class. A) Ribozyme class color bar for the number of RNAs in each subclass. B) bpRNA-CosMoS (fuzzy) clustering results for the Ribozyme class, showing multi-subclass cluster in the box. C) Structures from different subclasses that were clustered together.
